# Comparison of orthodontic tooth movement between adolescents and adults based on implant superimposition

**DOI:** 10.1371/journal.pone.0197281

**Published:** 2018-05-29

**Authors:** Meng-Jiao Ruan, Gui Chen, Tian-Min Xu

**Affiliations:** 1 Department of Orthodontics, Peking University School and Hospital of Stomatology, Beijing, China; 2 National Engineering Laboratory for Digital and Material Technology of Stomatology, Peking University School and Hospital of Stomatology, Beijing, China; 3 Beijing Key Laboratory of Digital Stomatology, Peking University School and Hospital of Stomatology, Beijing, China; Virginia Commonwealth University, UNITED STATES

## Abstract

**Objective:**

We compared tooth movement under maximum anchorage control with mini-screw implants in growing and non-growing patients.

**Methods:**

In total, 15 adolescent (G1) and 19 adult (G2) patients with prognathic profiles were selected. All patients underwent first premolar extraction treatment with mini-screw implants for maximum anchorage control. Cone-beam computed tomography (CBCT) data were obtained immediately after implant placement (T1) and at the end of anterior tooth retraction (T2). Tooth movement and root length changes of the maxillary first molar, canine, and incisors were evaluated with three-dimensional models constructed using CBCT data obtained before and after orthodontic retraction through the superimposition of stable implants.

**Results:**

Distal movement of the molar crown was observed in G2, but mesial movement was observed in G1. Mesial tipping of the first molar (1.82 ± 6.76°) was seen in G1 and distal tipping (4.44 ± 3.77°) was observed in G2. For the canines, mesial crown tipping (0.33 ± 4.99°) was noted in G1 and distal crown tipping (8.00 ± 5.57°) was observed in G2. In adults, the lingual inclinations of the lateral and central incisors were 11.91 ± 7.01° and 11.47 ± 6.70°, with 0.99 ± 1.22 mm and 1.08 ± 1.20 mm root retraction, respectively. In adolescents, the torque changes were smaller (lateral incisors, 8.25 ± 10.15°; central incisors, 9.82 ± 8.97°) and the root retractions were 0.31 ± 1.81 mm and 0.77 ± 1.59 mm, respectively. Less shortening of the central incisor roots occurred in adolescents than in adults.

**Conclusions:**

Tooth movements, such as anchor molar angular change, the canine tipping pattern, and the amount of incisor retraction, differed between adolescents and adults treated using the same anchorage with mini-screw implants, bracket prescription, and *en masse* retraction method. Anchorage strength of the first molars, canine movement patterns, and incisor retraction ranges are not determined by the anchorage device alone; growth and alveolar limitations also play roles.

## Introduction

Defined as resistance to unwanted tooth movement, orthodontic anchorage can be provided by other teeth, the palate, the head, the neck, or mini-screw implants. Among these, mini-screw implants undoubtedly provide the strongest anchorage, with no requirement for patient compliance[1,2]. Theoretically, they can keep a molar in its original position during treatment and retain the extraction space to relieve crowding or enable incisor retraction. However, studies of the anchorage strength of mini-screw implants in extraction treatment have produced inconsistent results. Benson et al.[3] and Sandler et al.[4] demonstrated significant anchorage losses, and Upadhyay et al.[5,6] reported distal movement and distal tipping. Thus, more evidence about implant anchorage effectiveness is needed.

Orthodontic anchorage control and tooth movement have been studied for decades, mainly by cephalometric analysis[3–9]. However, the limitations of radiographic cephalometric tracings, such as overlap of bilateral teeth and poor visualization of individual structures, cause errors in landmark identification and reduce measurement accuracy. Moreover, only the most protruded incisors and molars can be measured in such two-dimensional (2D) analyses, whereas the evaluation of canine movement during orthodontic treatment, especially in premolar extraction cases, is hardly possible.

Thiruvenkatachari et al. [10] reported that a three-dimensional (3D) laser scanner can be used to measure tooth displacement accurately and reliably, and that this approach can be considered as an alternative to the use of cephalometric radiographs. Lai et al.[11] demonstrated that skeletal anchorage achieved better results than did headgear in the treatment of maxillary dentoalveolar protrusion with 3D analyses of serial dental models. Park et al.[12] found less forward movement of the maxillary first molars in an implant group than in a conventional anchorage group. All studies conducted with 3D casts have concentrated on tooth crown movement; the roots were unobservable on the dental models.

Compared with dental casts, the 3D reconstruction and visualization of cone-beam computed tomography (CBCT) images can provide more detailed information on crown and root movements for each tooth. However, a problem with CBCT image analysis is the lack of a reliable and stable 3D region for superimposition. Liu et al.[13] investigated tooth movement in adult patients through landmark registration under the assumption that the shape of the upper skull was unchanged. For adolescents, such an assumption should be made cautiously because of maxillary growth[14]. The stability of implants has been researched; Chen and Nienkemper et al.[15,16] found that anchorage mini-screws stay nearly immobile, or at least show no clinically significant displacement compared with tooth movement, during orthodontic treatment. Miyawaki et al.[17] reported that implants with diameters > 1 mm placed in the buccal alveolar bone were more stable for orthodontic anchorage. Thus, superimposition on implants could be a valid way to evaluate tooth movement, especially in growing patients.

Orthodontists have preferred to treat adolescents, to work “with growth”[18–20], achieving better treatment results with greater efficiency. However, some studies have shown that anchorage is lost more in adolescents than in adults. Many orthodontists suspect that this difference may be related to compliance when headgear use, which depends on the patient[2]. From this perspective, adults usually have better self-control than do adolescents. However, whether this factor explains the difference is not clear.

In this study, because implant anchorage does not depend on patient compliance, we hypothesized that anchorage control strength and anterior tooth movement would not differ between adolescents and adults. We attempted to evaluate movement of the maxillary anterior teeth and first molar using 3D CBCT registration based on implants. We hope that the results provide useful clinical information for anchorage control and orthodontic treatment timing.

## Material and methods

### Orthodontic treatment and sample collection

The study sample was collected from Peking University School and Hospital of Stomatology, China. Inclusion criteria were: (1) Class I or II malocclusion with protrusive maxillary incisors (the value of U1/PP was larger than 115.8+5.7°or/and U1/SN larger than 105.7+6.3°based on the cephalometric measurements) and ≤3 mm crowding of the dentition, (2) indication for the extraction of bilateral maxillary first premolars and maximum anchorage control, (3) complete permanent dentition (not considering third molars), (4) good health with no chronic disease or disability, and (5) age 11–14 years for the adolescent group (G1) and >18 years for the adult group (G2). In total, 22 adolescents and 25 adults were enrolled. The adult samples were collected in our previous study from the year 2009 to 2011, and the adolescent samples were lately collected from the year 2013 to 2015. The research protocol was approved by the Ethics Committee of Peking University Biomedical Sciences, and all patients or guardians signed informed consent forms before enrollment.

Treatment was performed with straight wire appliances and 0.022-inch Roth prescription brackets (Xin Ya Corporation, Zhejiang, China). After initial leveling and alignment, the implants (1.6-mm diameter, 11-mm length; Ci Bei Corporation, Zhejiang, China) were placed under local anesthesia.

Six self-drilling mini-screws were placed in the maxilla of each adult participant. Two mini-screws inserted into the buccal inter-radicular space between the maxillary second premolar and first molar on both sides were used for *en masse* retraction of the anterior teeth, while four additional mini-screws placed in other inter-radicular spaces were not loaded. Unloaded mini-screws were inserted primarily between the lateral incisor and canine in the anterior region and between the first and second molars in the posterior region. The subjects have been described previously[15].

Loaded mini-screw implant positions were shown to be stable in our previous study[15]; thus, in the adolescent sample, only four mini-screws were placed in the maxilla. Two implants inserted into the buccal inter-radicular space between the maxillary first molar and second premolar on both sides, positioned in the region of the middle to apical third of the root, were used for *en masse* retraction of the anterior teeth. In the anterior region, two implants were placed in the labial inter-radicular space between the canine and lateral incisor or between the lateral incisor and central incisor on both sides to achieve vertical control of the anterior teeth during retraction, or they were kept unloaded.

*En masse* retraction of the anterior teeth against the implants was completed using a 0.019 × 0.025-inch stainless-steel straight wire and power chains with a force level of 150–250 g.

### CBCT data acquisition and processing

**Fig 1:**
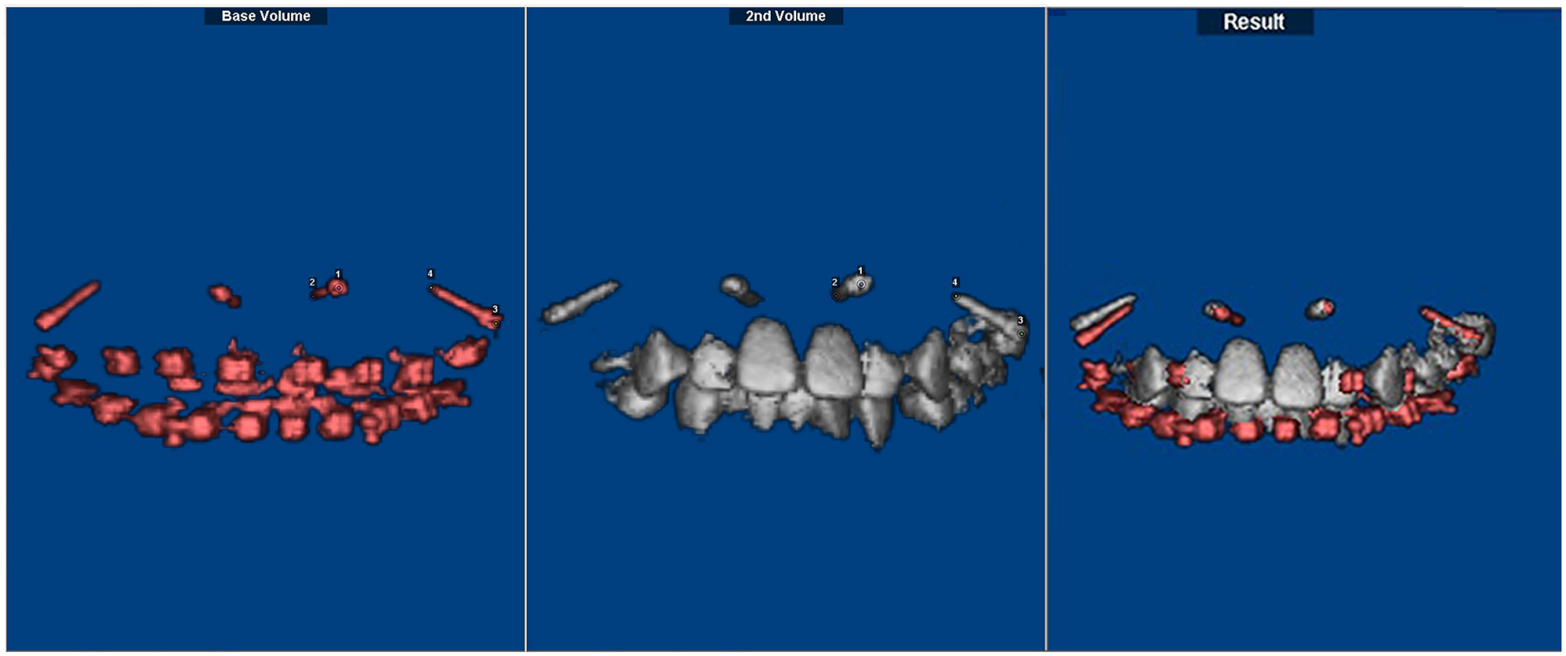
Implant superimposition for the adolescent sample. It was performed with the Dolphin software using the registration of four points: both endpoints of the two implants on the same side. Taking transverse palatal growth in adolescents into consideration, superimposition and measurement were processed on one side, and data from both sides were integrated.

**Fig 2:**
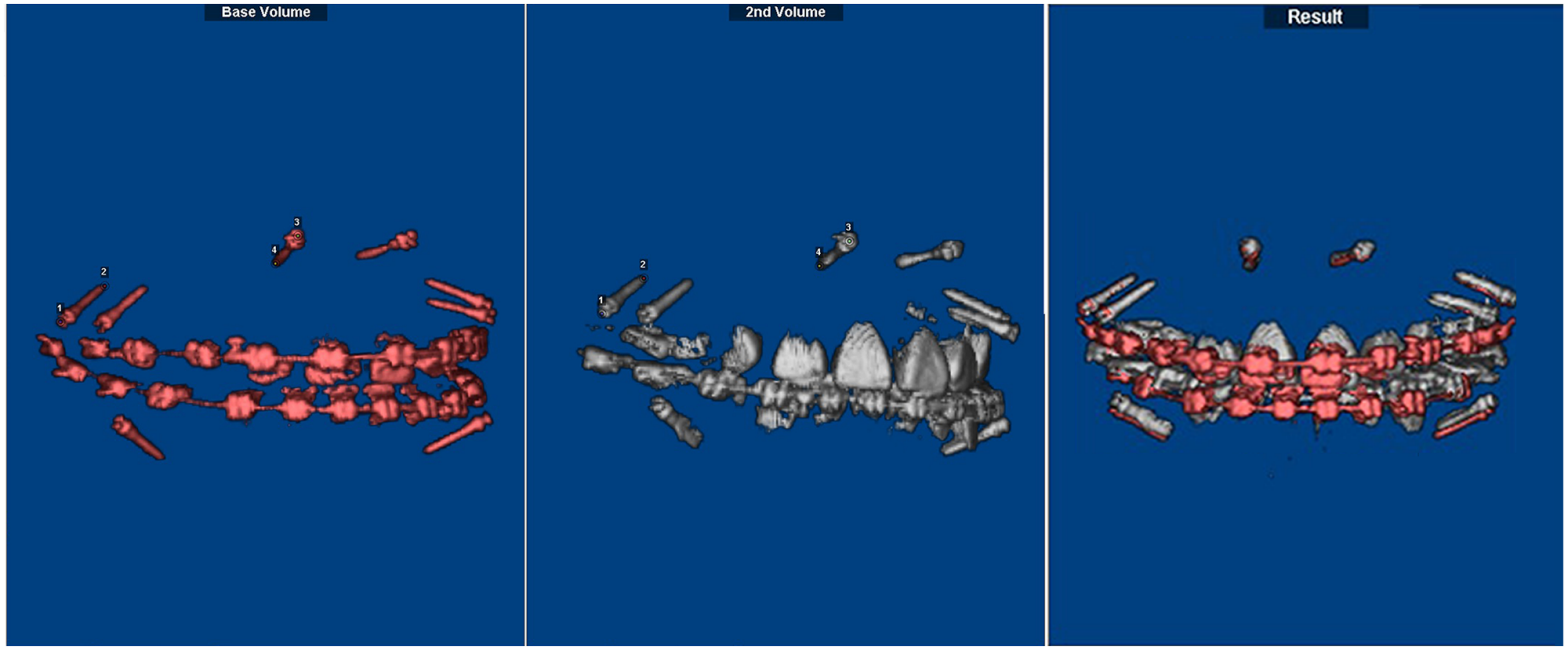
Implant superimposition for the adult sample. It was performed with the Dolphin software using the registration of four points: both endpoints of the two unloaded implants on the same side.

CBCT data were obtained immediately after implant placement (T1) and at the end of anterior tooth retraction (T2). The patients were positioned in centric occlusion with the lips closed and asked to remain still during the scanning procedure. Data were saved in digital imaging and communication in medicine (DICOM) format and entered into the Dolphin 3D software (Dolphin Imaging & Management Solutions Corp., USA) to accomplish superimposition of T1 and T2, which was done by the registration of four points: both endpoints of the two implants on the same side. In the adult sample, superimposition was achieved using unloaded implants in the posterior and anterior regions on the same side. In adolescents, both implants on the same side of the maxilla were used for superimposition (Figs 1 and 2).

**Fig 3:**
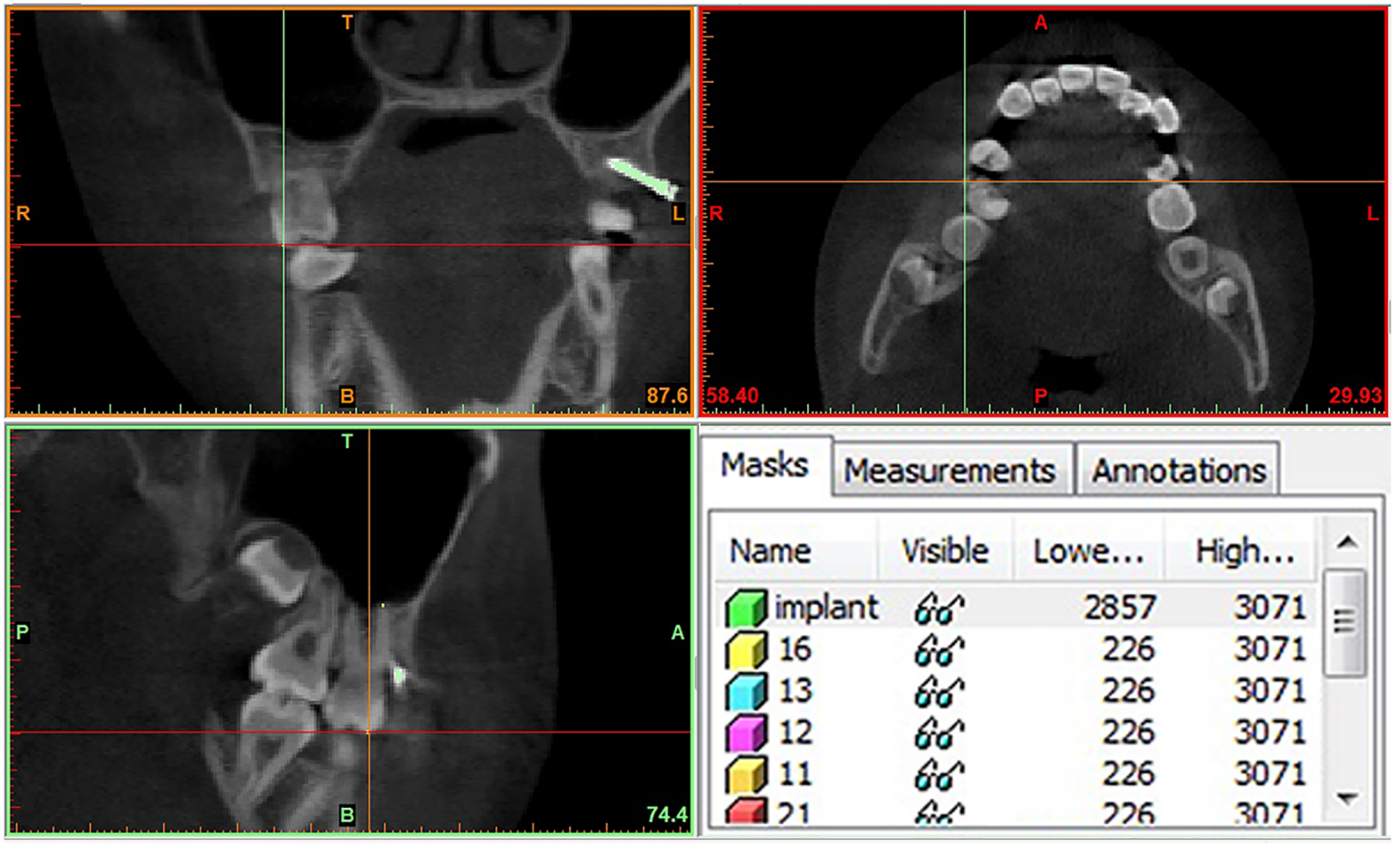
Segmentation of the metallic implants and tooth landmarks. Segmentation of the metallic implants from the maxilla and marking of tooth landmarks in the sagittal, coronal, and transverse planes. Separated masks of each tooth were created individually.

Then, the newly oriented volumes at the same coordinates were exported with no data loss. An interactive medical image control system (MIMICS 10.0; Materialise, Leuven, Belgium) was used to segment the metallic implants from the maxilla and mark the tooth points in the sagittal, coronal, and transverse planes at the same time (Fig 3). Separated masks of each tooth were created individually. This process allowed the generation of independent tooth files and 3D models. All 3D models were exported in standard triangulated language (STL) format.

### Method of measurement

Based on the scanned data, analyses of 3D models were performed using reverse engineering software (Rapidform2006; INUS Technology Inc., Seoul, Korea). A fitting transverse plane (plane 1) was built with points U6C and U1C on two sides of the T2 model. ANS’ and PNS’ were the projections of ANS and PNS on plane 1; thus, plane ANS-PNS-PNS’-ANS’ was defined as the sagittal plane (plane 2). A system of coordinates was established with PNS’ as the original point, and vector directions of PNS’-PNS (vertical) for X and PNS’-ANS’ (sagittal) for Y. Coordinate values for each tooth were exported for further linear calculations.

**Fig 4:**
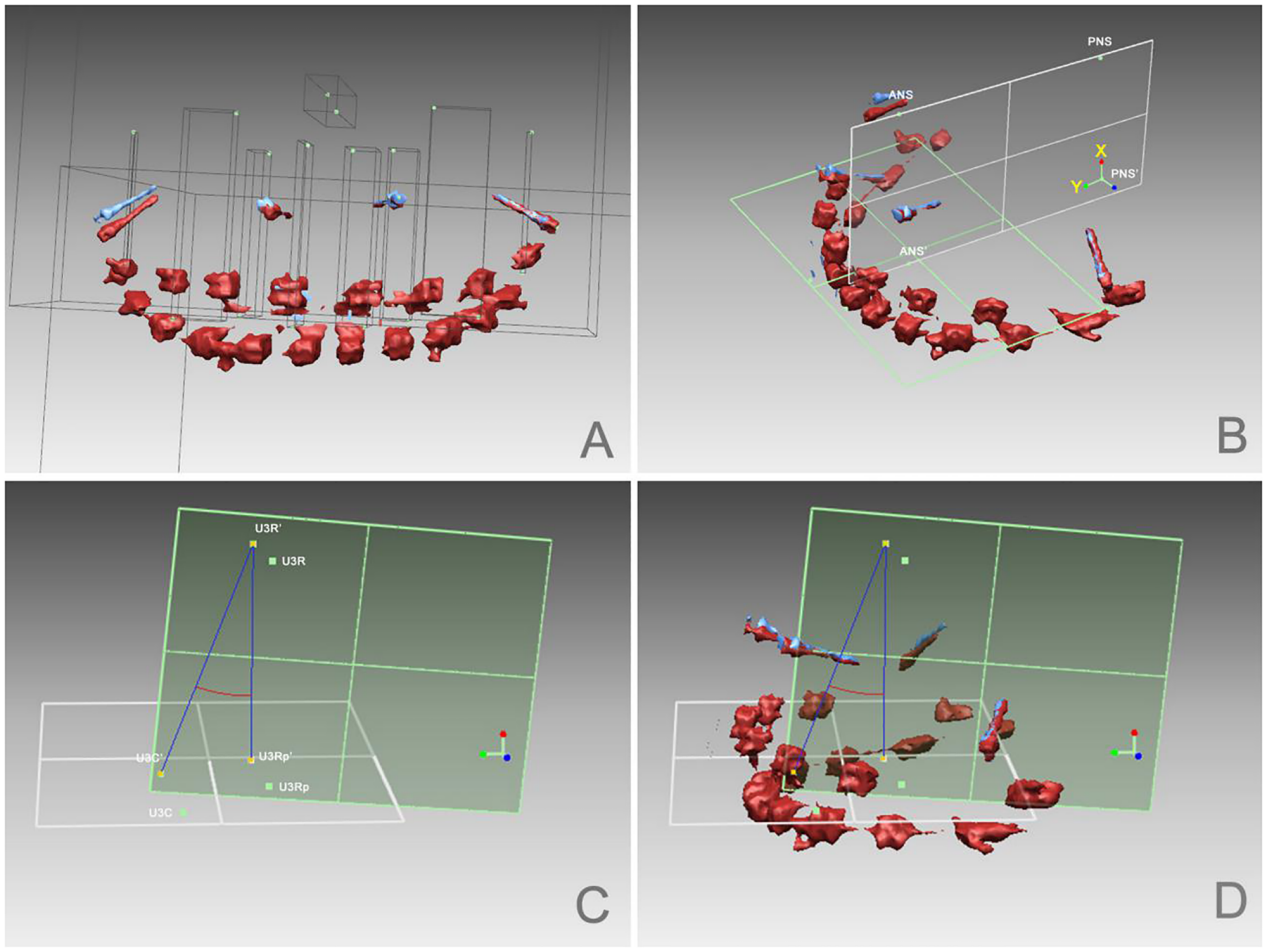
Analysis of 3D STL models performed using the Rapidform software. A. Landmarks were defined as follows: U6C, mesiobuccal crown cusp of the maxillary first molar; U6R, mesiobuccal root tip of the maxillary first molar; U3C, crown cusp of the maxillary canine; U3R, root tip of the maxillary canine; U2C, midpoint of the lateral incisor edge; U2R, root tip of the maxillary lateral incisor; U1C, midpoint of the central incisor edge; U1R, root tip of the maxillary central incisor; ANS, anterior nasal spine on model T2; and PNS, posterior nasal spine on model T2. B. A fitting transverse plane (plane 1) was built with points U6C and U1C on two sides of the T2 model; ANS-PNS-PNS’-ANS’ was defined as the sagittal plane (plane 2). C. U3Rp was the projection of U3R on plane 1, and U3C’, U3R’, and U3Rp’ were projections of U3C, U3R, and U3Rp on plane 2. D. Angle U3C’ U3R’ U3Rp’ was the sagittal angle of the canine.

To evaluate sagittal angle changes in molars and canines during incisor retraction, angular calculations were made. Projecting U3R and U6R onto plane 1 for U3Rp and U6Rp, U3C’, U3R’, and U3Rp’ and U6C’, U6R’, and U6Rp’ were the projections of U3C, U3R, and U3Rp, and U6C, U6R, and U6Rp on plane 2, respectively, and the angles of U3C’ U3R’ U3Rp’and U6C’ U6R’ U6Rp’ were the angles of the first molar and canine, respectively (Fig 4).

### Statistical analysis

Statistical analyses were performed using the SPSS software (SPSS for Windows, ver. 19.0; IBM SPSS, USA). For each variable measured on the 3D model, the mean and standard deviation were calculated. Paired-sample *t*-tests were used to evaluate changes between T1 and T2. Independent-sample *t*-tests were used for comparisons between the groups. Differences with probabilities less than 5% (*P* < 0.05) were considered to be statistically significant.

## Results

Of the 22 adolescents and 25 adults enrolled, 7 and 6 individuals, respectively, were excluded because of severe image blurring caused by metallic implants, patient motion, and/or implant loosening. This made the final sample sizes of these two groups were 15 adolescents (Male: 4; Female: 11) and 19 adults (Male: 6; Female 13). The average ages of each group were 12.5 and 24 years.

Before superimposition, implant positions, which have been shown to be stable in adults[15], were observed in G1 by measuring the endpoint distance between the two implants on the same side. This distance was reduced slightly at the “head” and “tail” points, but the differences were not significant (*t*-test; Table 1), indicating that the implants were stable with regard to each other and that we could use them for superimposition.

**Table 1:** Implant stability, determined by measuring the distances between the endpoints of two implants.

Measurement (mm)	T1		T2		T1 –T2		*P* value
	Mean	SD	Mean	SD	Mean	SD	
ImplantH	26.64	4.57	26.88	4.47	-0.24	0.58	0.07^n.s.^
ImplantT	18.75	3.8	18.95	3.81	-0.2	0.78	0.26 ^n.s.^

ImplantH: distance between the “head” points of the two implants; ImplantT: distance between the “tail” points of the two implants.

^n.s.^ No significant change (*P* > 0.05).

All variables were obtained by subtraction of the T2 values from the T1 values. Negative values indicated intrusion, mesial movement, or crown-anterior and root-posterior angular changes; positive values indicated extrusion, distal movement or incisor retraction, crown-posterior and root-anterior angular changes, or root resorption (Tables 2–4).

**Table 2:** Linear changes (T1—T2) in landmarks in the vertical (X) and mesiodistal (Y) directions.

Measurement (mm)	Vertical movement (X)					Mesiodistal movement (Y)				
	G1		G2		*P* value	G1		G2		*P* value
	Mean	SD	Mean	SD		Mean	SD	Mean	SD	
U6C	-0.29	1.01	-0.20	3.22	0.90	-0.97	2.10	0.73	1.64	0.00^†^
U6R	0.06	1.14	-1.02	3.08	0.11	-0.33	1.62	-0.37	1.39	0.93
U3C	0.15	1.29	0.61	1.42	0.21	4.47	1.79	5.00	2.62	0.40
U3R	0.35	1.62	-0.04	0.99	0.25	4.34	1.54	1.62	1.26	0.00^†^
U2C	0.27	1.64	1.13	1.42	0.04*	3.69	3.14	4.98	2.21	0.07
U2R	-0.13	1.96	-0.17	1.15	0.92	0.31	1.81	0.99	1.22	0.09
U1C	1.02	2.08	1.53	1.41	0.26	4.50	2.93	5.12	1.91	0.32
U1R	-0.64	1.76	0.11	0.87	0.07	0.77	1.59	1.08	1.20	0.40

*P < 0.05.

^†^*P* < 0.01.

**Table 3:** Displacement of the landmarks and root length changes for each tooth.

Measurement (mm)	G1		G2		*P* value
	Mean	SD	Mean	SD	
M6C	2.22	1.61	2.39	2.98	0.81
M6R	2.13	1.26	2.33	3.08	0.77
M3C	4.95	1.65	5.48	2.35	0.35
M3R	5.20	1.45	3.20	1.23	0.00^†^
M2C	5.09	1.90	5.39	2.22	0.60
M2R	2.74	1.34	2.19	0.97	0.07
M1C	5.58	2.07	5.60	1.93	0.97
M1R	2.77	1.15	1.80	0.81	0.00^†^
L6	0.71	1.11	0.42	0.69	0.26
L3	0.64	1.70	1.03	1.03	0.33
L2	0.96	1.41	1.04	1.33	0.83
L1	0.38	1.19	1.08	0.86	0.01^†^

M, landmark displacement; L, root length change.

^†^Significant difference between groups (*P* ≤ 0.01).

**Table 4:** Comparison of sagittal angle changes.

Measurement (°)	G1		G2		*P* value
	Mean	SD	Mean	SD	
Tip6	-1.82	6.76	4.44	3.77	< 0.01^†^
Tip3	-0.33	4.99	8.00	5.57	< 0.01^†^
Tor2	8.25	10.15	11.91	7.01	0.10
Tor1	9.82	8.97	11.47	6.70	0.42

^†^Significant difference between groups (*P* < 0.01).

1. **Maxillary first molar** (vertical movement). Vertical changes in first molar position were -0.29 ± 1.01 mm (U6C) and 0.06 ± 1.14 mm (U6R) in G1, and -0.20 ± 3.22 mm (U6C) and -1.02 ± 3.08 mm (U6R) in G2. Although with the mini-implants, a net intrusive effect on the molars was recorded in G1, it was not significant (*P* > 0.05; Table 2).
2. **Maxillary first molar** (sagittal movement). The changes in molar position during the retraction phase were -0.97 ± 2.10 mm (U6C) and -0.33 ± 1.62 mm (U6R) in G1, and 0.73 ± 1.64 mm (U6C) and -0.37 ± 1.39 mm (U6R) in G2. Molar crown movement was distal in G2, but mesial in G1; molar root apex movement was mesial in both groups. The difference in molar crown movement was significant (*P* ≤ 0.001; Table 2). In comparing the angular change, 1.82 ± 6.76° mesial inclination of the molar was observed in G1 and 4.44 ± 3.77° distal inclination of the molar was observed in G2; this difference was significant (Table 4).
3. **Maxillary canines**. Distal movement of the canines was noted in both groups, and a significant difference in canine root apex movement in the sagittal direction was found. The amounts of this distal movement were 4.34 ± 1.54 mm in G1 and 1.62 ± 1.26 mm in G2 (Table 2). Mesial crown tipping (0.33 ± 4.99°) was noted in G1, and distal crown tipping (8.00 ± 5.57°) was observed in G2 (*P* < 0.05; Table 4).
4. **Maxillary incisors**. Changes in incisor positions in both groups showed retraction, with positive values of T1 –T2 in the sagittal direction; no significant difference in the amount of incisor retraction was found between groups (*P* > 0.05). In adults, the lingual inclinations of lateral and central incisors were 11.91 ± 7.01° and 11.47 ± 6.70°, with 0.99 ± 1.22 mm and 1.08 ± 1.20 mm root retraction, respectively. In adolescents, the torque changes were lesser (lateral incisors, 8.25 ± 10.15°; central incisors, 9.82 ± 8.97°). The root retraction values were 0.31 ± 1.81 mm and 0.77 ± 1.59 mm, respectively (Tables 2 and 4). The differences in the displacement of incisors and tooth lengths were not significant, except for the root apex movement and length change of the central incisor. In G1, the central incisor root apex movement distance was 2.77 ± 1.15 mm, with length shortening (0.38 ± 1.19 mm); these values were 1.80 ± 0.81 mm and 1.08 ± 0.86 mm, respectively, in G2 (*P* < 0.05; Table 3).

## Discussion

### Molar movement and angular changes

Compared with the insignificant differences in molar intrusion and tooth length changes between the groups, sagittal movement of the molar warrants more attention. Distal tipping of the maxillary first molar (4.44 ± 3.77°) with mesial displacement of the root apex was observed in adults. This movement could be explained by the force of friction in sliding mechanics. When the anterior teeth were retracted with implant anchorage, the molar crown was pushed distally by friction force from the thick, rectangular stainless-steel wire. Similar results, obtained by 2D measurement of maxillary molar distalization during treatment with implants, were reported previously[7, 21, 22].

An interesting result was that the adolescent group showed molar tipping in the opposite direction (1.82 ± 6.76° mesially), with about the same amount of mesial movement of the root apex as in adults. The reasons for this difference can be considered from the following two points of view.

#### 1. Physiological factors in the patient

Björk and Skieller[23] stated that tooth position changed constantly to compensate for changes in jaw position during growth. Because the mandible usually grows more than the maxilla, to keep the molar relationship established, the maxillary molar moves forward approximately to cover the excess mandible, according to the recent study of Tsourakis and Johnston[24]. Another possible reason for this kind of anterior molar displacement may be the mesial component of occlusal force, commonly believed to cause the teeth to move toward the midline[25,26]. This physiological mesial movement caused by growth and/or occlusal force is not under the control of orthodontists and thus may lead to differences in molar angular change in adolescents and adults. This angulation change might also explain the vertical changes in the molar crown and root apex. In adults, the molar tipped distally, from a mesial inclination to upright; the root apex showed more intrusion than did the crown. In adolescents, intrusion of the crown and extrusion of the root apex, possible due to mesial tipping of the molar from a more upright status, were recorded.

#### 2. Prescription of appliance

Contemporary fixed appliances tend to use one prescription bracket, which is based on the Andrews’ “six keys standard”[27], to treat all malocclusions. Several studies have shown that the maxillary first molars are more distally tipped in adolescents than in adults[28–31]. Thus, the same 0° buccal tube on a maxillary molar may generate more mesial tipping in an adolescent than in an adult. Some studies have shown that more distal molar angulation before treatment tends to cause loss of anchorage to a greater extent in younger patients[32–35]. Jeremy et al.[36] observed more mesial tipping of the molar crowns in younger patients after orthodontic treatment with the Begg, edgewise, and straight-wire techniques, among which the molar tipped most mesially when using straight-wire appliances.

The observed difference in anchorage strength of the first molar between groups refuted our hypothesis. It indicates that the phenomenon of greater anchorage loss in adolescents is not solely because of compliance issues; growth in adolescents may play a role in this increased anchorage loss. New evidence of physiological anchorage loss during orthodontic treatment has been reported[37]; surprisingly, it was found that the physiological mesial movement of molars cannot be prevented completely, even with the use of mini-screw implants for anterior tooth retraction. This phenomenon may also explain the conflicting results on the efficacy of implant anchorage in previous reports[3–6].

### Canine movement and angular change

During anterior tooth retraction, the distal movements of the canine crown and root apex were 5.00 ± 2.62 mm and 1.62 ± 1.26 mm, respectively, in adults and 4.47 ± 1.79 mm and 4.34 ± 1.54 mm, respectively, in adolescents. Similar results for crown movement were reported by Lai et al.[11], who examined this issue using 3D digital casts, which prevented the observation of root apex displacement. A significant difference in the canine movement pattern was found between adolescents and adults. Bodily distal movement with 0.33 ± 4.99° mesial tipping was noted in adolescents, but apparent distal tipping of 8.00 ± 5.57° was found in adults, even with the same anchorage, bracket prescription, and *en masse* retraction method, indicating that distal movement of the canine root apex is easier in adolescents than in adults. To our knowledge, this difference has not been reported previously.

In panoramic film research, Bonetti et al.[38] found that the extent of mesial canine inclination increased between the ages of 8 and 9 years and decreased between the ages of 9 and 11 years. Fernandez et al.[39] also reported that the maxillary canine erupted with increasing mesial inclination, which reached a maximum at the age of 9 years, followed by gradual distal straightening. Coulter and Richardson[40] reported the occurrence of posterior movement in the maxillary canine crown between the ages of 7 and 13 years. In our study, the average age of the adolescents was 12.5 years, meaning that the canine was in the stage of physiological distal tipping, while the prescription brackets forced the canine root to tip distally. We suggest that the combined actions of the mechanical force of the appliances and physiological growth helped to realize the distal bodily movement of the canine in adolescents during treatment.

Also, in contrast to the mesial molar movement after treatment in adolescents, the molars of adults tipped distally, providing more space for distal canine tipping. While the difference in molar movement is attributable partly to physiological mesial movement of the dentition in adolescents, this result implies that not only the mechanical force of the appliances, but also the physiological mesial movement of the dentition in adolescents ultimately affected the position and angle of the canine after treatment. In previous studies based on the examination of 2D radiographs or 3D dental casts, the production of similar results regarding canine movement with comparison between adults and adolescents was hardly possible.

### Incisor movement and root resorption

The amount of incisor retraction observed in the two groups was comparable, with no significant difference. In G1, the central incisor root apex was displaced 2.77 ± 1.15 mm (0.77 ± 1.59 mm retraction and 0.64 ± 1.76 mm intrusion), with length shortening of 0.38 ± 1.19 mm, whereas the values in G2 were 1.80 ± 0.81 mm (1.08 ± 1.20 mm retraction and 0.11 ± 0.87 mm extrusion) and 1.08 ± 0.86 mm, respectively. In G2, the lingual inclinations of the lateral and central incisors were 11.91 ± 7.01° and 11.47 ± 6.70°, with 0.99 ± 1.22 mm and 1.08 ± 1.20 mm root retraction, respectively. In G1, the torque changes were smaller: 8.25 ± 10.15° for the lateral incisors and 9.82 ± 8.97° for the central incisors, with root retraction values of 0.31 ± 1.81 mm and 0.77 ± 1.59 mm, respectively.

More displacement of the incisor crown than the root apex was observed in both groups, suggesting that the achievement of bodily incisor retraction is difficult, even with such strong mini-screw implant anchorage, and that the physiological boundary of the alveolar bone also influences incisor movement. The larger incisor lingual inclination and root apex lingual movement observed in G2 may be attributable to more distal molar and canine tipping in adults, which may make more space for incisor retraction.

The observed extrusion of the incisor crown, but intrusion of the root apex, could be explained by inclination during retraction. Differences in incisor root apex displacement, the amount of which was underestimated by including root resorption, were not significant between groups, except for the central incisor root apex. The central incisor length change also differed significantly. The lengths of the central incisors were shortened less in adolescents than in adults.

Root resorption has been reported to be more obvious in adult patients than in children[41], and the combination of anterior retraction with intrusive mechanics has been found to cause more root resorption than does anterior retraction of the maxillary incisors alone[42,43]. Xu and Baumrind[44] found no significant correlation between mesiodistal movement of the root apex and root resorption in adolescents, whereas Baumrind et al.[45] reported a highly significant regression coefficient between incisor root resorption and the amount of root apex retraction. It was suggested that the immature root apex could move easily in developing alveolar bone, and that a lower resorption rate would result due to incomplete root calcification. Moreover, formation of the root itself might contribute to the lesser length change. One possibility is that orthodontic treatment, especially in those requiring maximum incisor retraction, should start early in adolescence, rather than in adulthood, considering tooth health issues.

Almost all correction depends on tooth movement in the treatment of non-growing adults[46,47], in contrast to treatment “with growth” in adolescents, and rates of complications, such as root resorption, may be higher. Furthermore, as tooth position changes constantly to compensate for changes in jaw position during growth[28,31], more mesial tipping of the dentition in adults leads to tooth movement patterns that differ from those in adolescents. The results of this study suggest that physiological mesial movement of the dentition in adolescents affects the position of and angular changes in teeth in orthodontic treatment.

## Conclusions

Tooth movement parameters, such as the anchorage strength of the first molars, canine tipping patterns, and the amount of incisor retraction, differed between groups, although the same anchorage with mini-screw implants, bracket prescription, and *en masse* retraction method were used. Mini-screw implants can achieve strong anchorage control in adolescents and can even push anchor molars distally in adults. The retraction of incisors is basically a tipping movement, with less root apex retraction, even with such strong anchorage. Canines showed bodily distal movement in adolescents, but apparent distal tipping in adults. More root resorption was observed in adults than in adolescents.

Anchorage strength for the first molars, canine movement patterns, and incisor retraction ranges are not determined by the anchorage device alone; growth and alveolar limitations also play roles. Orthodontic treatment, especially in those requiring maximum incisor retraction, should start early in adolescence, rather than in adulthood, considering tooth health issues.

## Supporting information

S1 Table Measurements of adult group. (DOCX)

S2 Table Measurements of adolescent group. (DOCX)

S1 STROBE checklist (PDF)
