# Supplementary material for: Comparison of orthodontic tooth movement between adolescents and adults based on implant superimposition
Source: PLoS One. 2018 May 29;13(5):e0197281. doi: 10.1371/journal.pone.0197281 (PMC5973581; doi:10.1371/journal.pone.0197281)
Supplement: S1 Table — (DOCX) [file pone.0197281.s001.docx]

S1 Table. Measurements of adult group

S1 Table A. X value of pre-treatment

|  | U6C | U6R | U3C | U3R | U2C | U2R | U1C | U1R |
| --- | --- | --- | --- | --- | --- | --- | --- | --- |
| 1 | 0.57 | 16.02 | -0.27 | 21.75 | 0.33 | 15.23 | 1.02 | 17.78 |
| 2 | 0.34 | 16.47 | -0.40 | 21.50 | 0.42 | 17.48 | 1.34 | 18.51 |
| 3 | -0.47 | 14.87 | -0.20 | 21.17 | -0.87 | 18.68 | -1.04 | 19.09 |
| 4 | -0.20 | 19.65 | -0.69 | 22.45 | 0.05 | 19.44 | 2.28 | 17.57 |
| 5 | 0.29 | 18.24 | 2.42 | 24.38 | 2.85 | 20.64 | 2.88 | 18.53 |
| 6 | 0.04 | 16.42 | -1.23 | 21.16 | -0.66 | 18.13 | -0.39 | 17.30 |
| 7 | -1.42 | 15.09 | 1.34 | 18.29 | 2.33 | 16.05 | 2.53 | 15.60 |
| 8 | -0.19 | 14.13 | 0.69 | 20.44 | 0.79 | 17.63 | 1.06 | 18.08 |
| 9 | 0.23 | 20.08 | 0.49 | 23.50 | 1.25 | 19.21 | 2.25 | 20.25 |
| 10 | 18.03 | -0.41 | 0.94 | 24.16 | 1.34 | 19.47 | 1.24 | 19.80 |
| 11 | -1.09 | 15.66 | 0.48 | 19.49 | 0.86 | 18.46 | 0.85 | 16.53 |
| 12 | -0.10 | 16.24 | 0.63 | 22.06 | 2.94 | 17.00 | 3.94 | 18.80 |
| 13 | -1.61 | 19.43 | -1.35 | 25.40 | 0.48 | 19.48 | -0.69 | 21.52 |
| 14 | -0.44 | 18.91 | 0.08 | 24.57 | 0.56 | 20.23 | 1.03 | 19.79 |
| 15 | -0.54 | 14.56 | -0.56 | 18.68 | 1.05 | 15.42 | 0.77 | 15.93 |
| 16 | -0.26 | 17.25 | 1.20 | 23.53 | 1.90 | 18.60 | 2.35 | 19.02 |
| 17 | -1.09 | 14.30 | 0.91 | 18.59 | 2.80 | 16.86 | 3.46 | 17.55 |
| 18 | -1.02 | 16.43 | 0.18 | 18.95 | 1.72 | 16.07 | 2.65 | 17.08 |
| 19 | 1.16 | 16.30 | 3.76 | 21.72 | 5.15 | 18.23 | 4.98 | 19.92 |
| 20 | -0.66 | 14.27 | -0.61 | 20.01 | 0.62 | 15.50 | 0.16 | 17.14 |
| 21 | -0.11 | 14.39 | -0.09 | 19.88 | 0.31 | 17.14 | 0.58 | 18.25 |
| 22 | -1.28 | 13.93 | -0.88 | 20.13 | -0.99 | 17.07 | -1.08 | 18.39 |
| 23 | -0.43 | 19.33 | -0.26 | 22.70 | 0.24 | 19.54 | 2.73 | 19.30 |
| 24 | -0.69 | 18.02 | 2.06 | 23.30 | 2.59 | 19.88 | 2.83 | 18.03 |
| 25 | -1.22 | 15.83 | -0.84 | 18.70 | -0.72 | 18.40 | -0.52 | 16.93 |
| 26 | 0.16 | 15.79 | 3.57 | 19.22 | 2.26 | 17.13 | 2.61 | 17.23 |
| 27 | -0.59 | 15.04 | -0.30 | 19.44 | 0.92 | 16.40 | 0.64 | 17.48 |
| 28 | 0.14 | 19.35 | 0.56 | 22.37 | 0.90 | 20.58 | 1.20 | 20.02 |
| 29 | -0.29 | 17.24 | 0.11 | 23.24 | 0.93 | 18.38 | 1.05 | 18.97 |
| 30 | -1.53 | 15.58 | 0.45 | 18.95 | 1.22 | 18.01 | 1.15 | 16.31 |
| 31 | -0.43 | 16.59 | 0.70 | 22.91 | 2.20 | 17.59 | 2.25 | 19.24 |
| 32 | -5.33 | 15.19 | -4.18 | 23.21 | -0.31 | 18.07 | -0.18 | 21.30 |
| 33 | -2.51 | 17.73 | 0.74 | 23.97 | 1.05 | 19.01 | 1.55 | 20.24 |
| 34 | -1.06 | 14.94 | 0.14 | 19.96 | 0.78 | 14.73 | 0.44 | 15.03 |
| 35 | -0.61 | 17.77 | 0.72 | 24.93 | 1.79 | 18.29 | 1.84 | 18.09 |
| 36 | -1.40 | 14.16 | 1.39 | 17.81 | 3.12 | 16.32 | 2.84 | 17.66 |
| 37 | -0.12 | 17.26 | 0.36 | 19.86 | 1.80 | 17.91 | 2.27 | 17.65 |
| 38 | -0.18 | 14.58 | 2.07 | 21.24 | 3.69 | 17.96 | 4.48 | 19.36 |

S1 Table B. Y value of pre-treatment

|  | U6C | U6R | U3C | U3R | U2C | U2R | U1C | U1R |
| --- | --- | --- | --- | --- | --- | --- | --- | --- |
| 1 | 31.87 | 25.85 | 48.51 | 37.15 | 54.96 | 43.92 | 58.70 | 46.28 |
| 2 | 32.10 | 27.38 | 42.68 | 36.96 | 48.93 | 40.48 | 51.93 | 42.98 |
| 3 | 30.55 | 26.07 | 46.05 | 39.05 | 52.05 | 44.59 | 55.64 | 47.66 |
| 4 | 29.13 | 24.43 | 48.34 | 38.42 | 53.80 | 42.14 | 60.07 | 44.06 |
| 5 | 27.94 | 20.34 | 48.95 | 34.43 | 55.20 | 40.90 | 58.31 | 44.20 |
| 6 | 31.11 | 27.44 | 52.40 | 45.32 | 59.61 | 47.63 | 64.29 | 52.65 |
| 7 | 28.11 | 22.57 | 48.28 | 34.95 | 50.00 | 39.94 | 54.87 | 42.43 |
| 8 | 28.36 | 24.04 | 44.25 | 32.74 | 50.39 | 39.52 | 53.13 | 41.38 |
| 9 | 28.44 | 22.51 | 44.56 | 36.97 | 51.65 | 42.01 | 55.55 | 43.31 |
| 10 | 24.37 | 30.58 | 48.55 | 35.37 | 54.66 | 41.06 | 57.89 | 43.91 |
| 11 | 32.03 | 26.14 | 49.64 | 37.54 | 54.55 | 43.09 | 56.84 | 47.24 |
| 12 | 31.45 | 27.43 | 53.46 | 41.82 | 60.67 | 46.89 | 65.27 | 47.91 |
| 13 | 30.78 | 26.71 | 51.55 | 38.50 | 58.66 | 43.68 | 61.37 | 45.97 |
| 14 | 30.37 | 25.96 | 52.77 | 39.65 | 58.61 | 43.35 | 63.62 | 48.33 |
| 15 | 29.50 | 25.46 | 50.12 | 40.10 | 57.13 | 47.35 | 59.55 | 48.79 |
| 16 | 31.84 | 24.42 | 50.52 | 37.89 | 57.27 | 45.58 | 61.12 | 49.30 |
| 17 | 31.04 | 26.81 | 47.69 | 36.99 | 53.69 | 41.73 | 56.86 | 42.47 |
| 18 | 27.36 | 23.01 | 48.33 | 35.64 | 55.08 | 42.34 | 58.74 | 44.32 |
| 19 | 33.04 | 26.73 | 52.29 | 36.94 | 58.70 | 43.92 | 62.12 | 46.15 |
| 20 | 32.34 | 27.66 | 49.53 | 37.88 | 55.42 | 43.39 | 59.39 | 46.10 |
| 21 | 32.17 | 29.87 | 44.09 | 35.80 | 49.06 | 40.09 | 51.98 | 42.58 |
| 22 | 31.82 | 26.82 | 48.05 | 37.98 | 53.00 | 45.43 | 55.88 | 47.36 |
| 23 | 28.58 | 23.47 | 48.03 | 36.38 | 53.73 | 42.35 | 59.24 | 43.48 |
| 24 | 29.63 | 22.93 | 51.17 | 36.48 | 56.47 | 42.85 | 59.05 | 45.24 |
| 25 | 32.28 | 27.27 | 52.88 | 45.46 | 60.00 | 47.56 | 64.43 | 51.62 |
| 26 | 26.99 | 19.81 | 46.93 | 34.21 | 49.19 | 39.20 | 54.44 | 40.31 |
| 27 | 31.52 | 26.18 | 44.59 | 33.71 | 51.81 | 38.38 | 53.90 | 41.33 |
| 28 | 26.78 | 23.08 | 44.58 | 36.45 | 51.17 | 41.49 | 55.39 | 43.65 |
| 29 | 30.50 | 26.01 | 48.00 | 36.30 | 53.61 | 40.01 | 58.34 | 43.56 |
| 30 | 31.14 | 26.52 | 49.37 | 37.84 | 54.34 | 42.54 | 57.26 | 47.67 |
| 31 | 33.20 | 30.78 | 54.45 | 39.28 | 60.83 | 44.54 | 64.55 | 47.77 |
| 32 | 31.60 | 26.94 | 52.00 | 39.58 | 58.04 | 46.64 | 61.56 | 45.35 |
| 33 | 30.87 | 25.34 | 52.91 | 38.59 | 58.81 | 43.50 | 63.84 | 46.14 |
| 34 | 27.79 | 22.66 | 48.76 | 37.47 | 56.15 | 46.32 | 59.72 | 48.79 |
| 35 | 34.29 | 28.21 | 50.84 | 38.56 | 58.02 | 46.98 | 61.56 | 49.17 |
| 36 | 29.71 | 25.23 | 48.42 | 38.07 | 53.54 | 41.46 | 56.93 | 42.47 |
| 37 | 30.69 | 27.07 | 49.12 | 36.09 | 55.12 | 42.35 | 59.11 | 43.79 |
| 38 | 34.64 | 28.80 | 53.01 | 38.02 | 58.48 | 44.15 | 61.82 | 46.16 |

S1 Table C. X value of post-treatment

|  | U6C | U6R | U3C | U3R | U2C | U2R | U1C | U1R |
| --- | --- | --- | --- | --- | --- | --- | --- | --- |
| 1 | -0.07 | 16.27 | -0.54 | 20.76 | -0.05 | 16.31 | 0.38 | 16.39 |
| 2 | -0.13 | 15.01 | -0.30 | 19.99 | 0.69 | 16.82 | 0.80 | 17.71 |
| 3 | 0.02 | 15.93 | -0.36 | 19.80 | 0.19 | 16.59 | -0.10 | 18.78 |
| 4 | 0.04 | 19.30 | -0.34 | 22.47 | -0.25 | 19.60 | -0.23 | 18.47 |
| 5 | -0.02 | 18.06 | -0.28 | 23.36 | 0.49 | 19.67 | 0.10 | 19.10 |
| 6 | -0.09 | 15.56 | 0.67 | 21.70 | 0.99 | 19.47 | 0.61 | 16.66 |
| 7 | 0.01 | 14.55 | 0.00 | 17.96 | 0.24 | 14.39 | -0.03 | 15.69 |
| 8 | -0.20 | 14.31 | -0.20 | 19.20 | 0.69 | 17.41 | 0.97 | 17.83 |
| 9 | 0.00 | 19.07 | -1.34 | 23.12 | -0.34 | 19.70 | 0.01 | 20.62 |
| 10 | -0.01 | 18.31 | -0.94 | 23.11 | 0.04 | 20.17 | 0.09 | 20.59 |
| 11 | 0.02 | 17.71 | -0.47 | 19.17 | 0.21 | 19.47 | -0.11 | 14.90 |
| 12 | -0.03 | 16.11 | -1.50 | 23.35 | -1.18 | 18.91 | 0.15 | 20.25 |
| 13 | 0.03 | 20.65 | 0.30 | 25.73 | 1.30 | 19.78 | -0.22 | 21.37 |
| 14 | -0.08 | 19.38 | -0.08 | 26.50 | 0.39 | 22.15 | 0.44 | 20.37 |
| 15 | -0.06 | 16.03 | -0.35 | 20.90 | -0.09 | 15.51 | 0.31 | 15.54 |
| 16 | 0.05 | 17.96 | 0.21 | 22.89 | 0.29 | 20.08 | -0.26 | 18.47 |
| 17 | -0.01 | 14.76 | -0.04 | 18.50 | 0.24 | 17.77 | 0.06 | 18.37 |
| 18 | 0.01 | 16.55 | -0.87 | 20.26 | 0.17 | 16.10 | -0.06 | 17.94 |
| 19 | 0.43 | 16.68 | 0.34 | 22.90 | 0.94 | 17.57 | 0.85 | 18.74 |
| 20 | 0.07 | 14.78 | -0.33 | 20.85 | -0.14 | 15.84 | -0.38 | 15.04 |
| 21 | 0.17 | 15.37 | -0.62 | 19.55 | -0.58 | 16.16 | -0.84 | 17.68 |
| 22 | -0.02 | 15.03 | 0.22 | 20.36 | 0.05 | 15.61 | 0.10 | 19.54 |
| 23 | -0.04 | 19.51 | -0.18 | 21.95 | 0.06 | 19.77 | 0.22 | 19.25 |
| 24 | 0.02 | 18.33 | 0.20 | 22.27 | -0.11 | 18.50 | -0.09 | 17.50 |
| 25 | 0.12 | 16.53 | 0.99 | 18.76 | -0.77 | 18.23 | -0.64 | 16.07 |
| 26 | -0.01 | 16.10 | -0.18 | 17.62 | 0.29 | 15.03 | 0.03 | 16.92 |
| 27 | 0.16 | 15.53 | -0.42 | 19.77 | -0.50 | 17.06 | -0.93 | 17.37 |
| 28 | 0.00 | 19.56 | -0.70 | 21.74 | 0.42 | 20.88 | -0.01 | 20.04 |
| 29 | 0.02 | 17.82 | -0.78 | 23.45 | 0.07 | 19.57 | -0.09 | 19.74 |
| 30 | -0.02 | 16.69 | -0.51 | 18.60 | 0.34 | 17.91 | 0.11 | 14.61 |
| 31 | 0.03 | 17.38 | 0.27 | 23.87 | -0.37 | 18.08 | -0.15 | 20.10 |
| 32 | -0.03 | 18.73 | -1.20 | 23.81 | 0.74 | 19.95 | 0.21 | 21.44 |
| 33 | 0.09 | 20.05 | 0.73 | 25.91 | 0.41 | 21.42 | -0.45 | 20.97 |
| 34 | 0.04 | 14.99 | 0.56 | 19.71 | 0.05 | 13.89 | -0.30 | 14.63 |
| 35 | -0.04 | 18.00 | 0.20 | 24.91 | -0.14 | 18.79 | 0.26 | 17.73 |
| 36 | 0.01 | 15.18 | 0.30 | 18.54 | 0.40 | 17.88 | -0.06 | 17.79 |
| 37 | -0.01 | 17.69 | -0.99 | 20.93 | -0.03 | 17.74 | 0.06 | 18.71 |
| 38 | 1.24 | 16.06 | -0.50 | 20.80 | -0.26 | 17.01 | 0.44 | 18.17 |

S1 Table D. Y value of post-treatment

|  | U6C | U6R | U3C | U3R | U2C | U2R | U1C | U1R |
| --- | --- | --- | --- | --- | --- | --- | --- | --- |
| 1 | 29.05 | 26.27 | 42.36 | 34.89 | 49.92 | 42.57 | 53.80 | 43.98 |
| 2 | 32.55 | 28.30 | 43.63 | 37.32 | 48.23 | 40.55 | 51.11 | 42.51 |
| 3 | 28.90 | 26.68 | 44.57 | 37.56 | 50.47 | 44.48 | 53.41 | 46.20 |
| 4 | 29.01 | 24.22 | 48.33 | 38.53 | 53.89 | 42.72 | 56.97 | 44.74 |
| 5 | 24.89 | 20.55 | 39.61 | 30.01 | 47.47 | 40.10 | 51.10 | 43.53 |
| 6 | 31.70 | 29.04 | 46.99 | 41.88 | 55.18 | 46.65 | 59.92 | 49.73 |
| 7 | 26.84 | 22.48 | 40.86 | 32.67 | 45.59 | 40.50 | 48.50 | 42.04 |
| 8 | 31.64 | 25.88 | 43.36 | 33.70 | 48.25 | 40.04 | 50.85 | 41.28 |
| 9 | 25.99 | 23.95 | 39.69 | 35.10 | 46.12 | 41.33 | 49.91 | 43.87 |
| 10 | 30.32 | 24.41 | 44.97 | 34.53 | 49.62 | 42.01 | 53.62 | 43.37 |
| 11 | 31.22 | 27.94 | 45.58 | 36.75 | 48.99 | 42.92 | 52.11 | 44.67 |
| 12 | 29.72 | 27.25 | 47.77 | 39.91 | 54.34 | 43.77 | 58.81 | 47.23 |
| 13 | 30.38 | 28.88 | 46.88 | 35.66 | 51.79 | 42.87 | 56.71 | 43.85 |
| 14 | 30.34 | 25.27 | 45.94 | 37.80 | 52.60 | 42.67 | 57.48 | 45.77 |
| 15 | 29.11 | 26.58 | 44.29 | 36.52 | 50.93 | 44.77 | 54.90 | 46.45 |
| 16 | 31.35 | 25.49 | 44.99 | 35.78 | 51.64 | 43.76 | 55.59 | 48.55 |
| 17 | 28.66 | 26.98 | 41.42 | 33.55 | 46.69 | 39.94 | 49.39 | 39.50 |
| 18 | 27.05 | 23.55 | 43.51 | 35.23 | 49.04 | 39.95 | 52.00 | 42.55 |
| 19 | 31.61 | 26.48 | 43.15 | 36.01 | 48.84 | 44.12 | 53.63 | 44.81 |
| 20 | 31.40 | 27.21 | 43.94 | 36.03 | 50.53 | 40.60 | 54.36 | 44.65 |
| 21 | 33.02 | 31.21 | 43.86 | 36.72 | 48.13 | 40.38 | 50.32 | 42.37 |
| 22 | 30.90 | 27.95 | 45.42 | 36.16 | 50.69 | 43.96 | 53.51 | 46.27 |
| 23 | 28.42 | 23.43 | 47.39 | 36.27 | 53.28 | 42.11 | 56.96 | 45.06 |
| 24 | 29.28 | 23.67 | 41.97 | 34.25 | 49.98 | 42.82 | 52.98 | 45.71 |
| 25 | 32.05 | 28.26 | 47.17 | 42.73 | 55.12 | 45.84 | 58.60 | 48.66 |
| 26 | 25.95 | 20.64 | 38.59 | 32.83 | 44.29 | 38.66 | 47.98 | 41.24 |
| 27 | 29.25 | 26.46 | 42.29 | 32.75 | 47.67 | 38.89 | 51.25 | 42.00 |
| 28 | 26.11 | 22.77 | 39.31 | 33.39 | 45.24 | 41.01 | 49.61 | 42.50 |
| 29 | 29.59 | 26.97 | 44.31 | 34.92 | 48.68 | 40.03 | 52.47 | 43.75 |
| 30 | 30.82 | 27.07 | 43.19 | 36.27 | 48.65 | 40.22 | 51.86 | 44.90 |
| 31 | 29.85 | 29.65 | 47.44 | 38.60 | 55.33 | 42.98 | 58.74 | 47.65 |
| 32 | 30.74 | 26.44 | 46.46 | 36.36 | 53.75 | 42.11 | 56.99 | 43.88 |
| 33 | 30.10 | 27.12 | 45.54 | 36.58 | 52.04 | 42.99 | 56.80 | 44.18 |
| 34 | 27.30 | 25.10 | 43.97 | 35.65 | 51.85 | 45.37 | 56.29 | 47.37 |
| 35 | 32.86 | 27.71 | 45.77 | 37.72 | 51.61 | 46.44 | 56.03 | 48.59 |
| 36 | 28.42 | 25.10 | 40.56 | 35.31 | 45.94 | 39.21 | 49.03 | 40.25 |
| 37 | 28.97 | 26.84 | 44.94 | 35.29 | 50.37 | 40.28 | 52.64 | 42.23 |
| 38 | 32.77 | 28.67 | 45.72 | 37.67 | 50.48 | 42.27 | 53.55 | 44.98 |

S1 Table E. Root length of pre/post-treatment

|  | Pre-treatment | | | |  | Post-treatment | | | |
| --- | --- | --- | --- | --- | --- | --- | --- | --- | --- |
|  | U6 | U3 | U2 | U1 |  | U6 | U3 | U2 | U1 |
| 1 | 16.60 | 24.90 | 18.87 | 20.99 |  | 16.89 | 22.58 | 18.02 | 18.82 |
| 2 | 16.88 | 22.70 | 19.06 | 19.37 |  | 15.82 | 21.26 | 17.89 | 18.99 |
| 3 | 16.06 | 22.58 | 21.21 | 21.68 |  | 16.08 | 21.35 | 17.48 | 20.29 |
| 4 | 20.41 | 25.28 | 22.77 | 22.14 |  | 19.89 | 25.13 | 23.14 | 22.35 |
| 5 | 19.50 | 26.65 | 23.94 | 21.20 |  | 18.64 | 25.51 | 21.19 | 20.57 |
| 6 | 16.84 | 23.49 | 22.85 | 21.23 |  | 16.13 | 21.79 | 20.71 | 19.02 |
| 7 | 17.52 | 22.14 | 17.08 | 18.06 |  | 15.47 | 19.86 | 15.08 | 17.00 |
| 8 | 15.03 | 23.58 | 20.66 | 20.76 |  | 15.65 | 21.69 | 18.79 | 19.40 |
| 9 | 21.20 | 24.46 | 21.06 | 21.86 |  | 19.29 | 24.90 | 21.08 | 21.48 |
| 10 | 19.58 | 27.04 | 23.21 | 23.35 |  | 19.32 | 26.24 | 22.05 | 22.94 |
| 11 | 17.76 | 22.62 | 21.14 | 18.43 |  | 18.21 | 21.57 | 20.26 | 16.78 |
| 12 | 16.87 | 24.77 | 20.34 | 23.01 |  | 16.39 | 26.23 | 23.33 | 23.29 |
| 13 | 21.44 | 29.80 | 24.49 | 27.04 |  | 20.68 | 27.83 | 20.62 | 25.13 |
| 14 | 19.87 | 27.81 | 25.37 | 24.31 |  | 20.13 | 27.82 | 24.14 | 23.20 |
| 15 | 15.63 | 22.11 | 17.96 | 18.59 |  | 16.33 | 22.93 | 17.47 | 17.53 |
| 16 | 19.03 | 25.99 | 20.68 | 20.56 |  | 19.26 | 24.49 | 21.35 | 20.07 |
| 17 | 16.01 | 20.84 | 19.20 | 20.22 |  | 15.01 | 20.16 | 19.11 | 20.84 |
| 18 | 17.99 | 23.00 | 19.49 | 20.43 |  | 17.11 | 22.75 | 18.38 | 20.34 |
| 19 | 16.61 | 24.75 | 20.42 | 22.12 |  | 17.12 | 23.70 | 17.56 | 20.04 |
| 20 | 15.66 | 23.87 | 19.23 | 21.58 |  | 15.59 | 22.64 | 18.84 | 18.24 |
| 21 | 14.68 | 21.62 | 19.09 | 20.03 |  | 15.36 | 21.40 | 18.48 | 20.16 |
| 22 | 16.01 | 23.33 | 19.65 | 21.26 |  | 15.39 | 22.17 | 16.95 | 20.78 |
| 23 | 20.41 | 25.83 | 22.55 | 22.87 |  | 20.18 | 25.29 | 22.93 | 22.49 |
| 24 | 19.87 | 25.83 | 22.10 | 20.56 |  | 19.21 | 23.42 | 19.96 | 19.06 |
| 25 | 17.84 | 20.98 | 22.95 | 21.67 |  | 17.08 | 18.42 | 21.27 | 19.45 |
| 26 | 17.20 | 20.81 | 18.21 | 20.34 |  | 17.05 | 18.73 | 15.82 | 18.18 |
| 27 | 16.52 | 22.96 | 20.87 | 21.02 |  | 15.72 | 23.23 | 20.03 | 20.55 |
| 28 | 19.57 | 23.31 | 22.10 | 22.25 |  | 19.86 | 23.21 | 21.23 | 21.32 |
| 29 | 18.26 | 25.99 | 22.90 | 23.24 |  | 17.99 | 26.01 | 21.88 | 21.67 |
| 30 | 17.72 | 21.97 | 20.60 | 17.95 |  | 17.42 | 20.32 | 19.55 | 16.10 |
| 31 | 17.19 | 27.04 | 22.70 | 23.91 |  | 17.36 | 25.20 | 22.51 | 23.08 |
| 32 | 21.04 | 30.09 | 21.67 | 26.91 |  | 19.31 | 27.00 | 22.67 | 24.95 |
| 33 | 21.10 | 27.34 | 24.02 | 25.74 |  | 20.20 | 26.74 | 23.22 | 24.90 |
| 34 | 16.86 | 22.96 | 17.43 | 18.34 |  | 15.50 | 20.90 | 15.42 | 17.42 |
| 35 | 19.38 | 27.27 | 20.23 | 20.44 |  | 18.81 | 26.00 | 19.68 | 19.00 |
| 36 | 16.20 | 19.56 | 18.75 | 20.74 |  | 15.53 | 19.00 | 19.04 | 19.91 |
| 37 | 17.83 | 23.66 | 21.02 | 21.74 |  | 17.97 | 23.98 | 20.46 | 21.36 |
| 38 | 15.91 | 24.39 | 20.38 | 21.61 |  | 15.40 | 22.84 | 19.14 | 19.69 |
