# Supplementary material for: Comparison of orthodontic tooth movement between adolescents and adults based on implant superimposition
Source: PLoS One. 2018 May 29;13(5):e0197281. doi: 10.1371/journal.pone.0197281 (PMC5973581; doi:10.1371/journal.pone.0197281)
Supplement: S2 Table — (DOCX) [file pone.0197281.s002.docx]

S2 Table. Measurements of adolescent group

S2 Table A. X value of pre-treatment

|  | U6C | U6R | U3C | U3R | U2C | U2R | U1C | U1R |
| --- | --- | --- | --- | --- | --- | --- | --- | --- |
| 1 | 0.02 | 17.78 | -1.55 | 22.30 | -0.35 | 18.86 | -1.55 | 20.12 |
| 2 | -0.41 | 18.82 | 1.29 | 24.14 | 3.11 | 21.66 | 3.99 | 23.31 |
| 3 | -0.91 | 14.11 | 0.61 | 18.39 | 1.34 | 14.95 | 2.93 | 15.88 |
| 4 | -0.99 | 15.33 | -0.55 | 20.39 | -0.08 | 16.99 | 0.90 | 17.26 |
| 5 | -0.72 | 15.54 | -1.13 | 20.74 | 0.74 | 19.15 | 0.56 | 19.62 |
| 6 | -0.08 | 17.13 | -1.32 | 22.28 | 0.17 | 17.76 | 0.37 | 18.97 |
| 7 | 2.76 | 23.52 | -0.92 | 21.72 | -2.31 | 17.78 | -2.53 | 19.65 |
| 8 | -1.16 | 16.97 | -0.96 | 22.25 | -0.75 | 18.73 | -1.05 | 17.71 |
| 9 | 0.01 | 18.94 | 2.77 | 24.10 | 4.53 | 19.39 | 5.66 | 20.41 |
| 10 | 0.93 | 18.85 | -0.39 | 22.12 | 0.51 | 19.02 | 0.04 | 15.21 |
| 11 | -1.31 | 16.79 | -1.47 | 20.29 | 0.06 | 15.77 | 0.36 | 14.91 |
| 12 | -0.74 | 17.64 | -1.79 | 22.77 | -0.39 | 18.24 | -0.21 | 20.98 |
| 13 | 0.55 | 18.15 | 1.59 | 24.91 | 3.64 | 22.19 | 4.46 | 22.27 |
| 14 | -1.44 | 13.79 | 0.74 | 18.95 | 2.95 | 15.27 | 2.70 | 17.33 |
| 15 | -1.53 | 16.32 | 0.33 | 22.46 | 1.23 | 17.08 | 0.76 | 18.17 |
| 16 | -1.25 | 14.90 | -1.47 | 20.52 | 0.63 | 17.50 | -0.29 | 17.87 |
| 17 | 0.33 | 18.21 | -0.79 | 23.66 | -0.45 | 18.48 | 0.41 | 18.89 |
| 18 | 0.81 | 19.85 | 0.70 | 19.92 | -0.78 | 16.63 | -0.83 | 16.13 |
| 19 | 0.38 | 19.10 | 0.93 | 24.54 | 0.89 | 17.22 | 1.27 | 18.96 |
| 20 | -1.51 | 16.72 | -0.26 | 22.89 | -1.28 | 17.88 | 0.46 | 17.16 |
| 21 | 0.57 | 18.94 | 1.75 | 24.44 | 3.43 | 20.00 | 4.23 | 20.83 |
| 22 | -0.35 | 18.28 | -0.62 | 22.80 | -0.69 | 18.59 | -0.22 | 15.46 |
| 23 | -0.39 | 17.56 | -1.54 | 21.72 | -0.52 | 17.40 | 0.63 | 17.43 |

S2 Table B. Y value of pre-treatment

|  | U6C | U6R | U3C | U3R | U2C | U2R | U1C | U1R |
| --- | --- | --- | --- | --- | --- | --- | --- | --- |
| 1 | 30.61 | 28.17 | 50.03 | 41.20 | 56.60 | 44.97 | 59.70 | 46.68 |
| 2 | 28.71 | 24.26 | 46.87 | 31.97 | 51.72 | 35.00 | 54.97 | 35.53 |
| 3 | 28.32 | 24.32 | 49.64 | 34.25 | 52.52 | 39.26 | 59.26 | 43.30 |
| 4 | 30.20 | 26.06 | 51.14 | 40.36 | 57.28 | 45.56 | 62.05 | 46.41 |
| 5 | 25.52 | 23.69 | 46.80 | 38.96 | 52.15 | 39.45 | 57.88 | 43.02 |
| 6 | 15.96 | 13.90 | 36.79 | 26.56 | 40.59 | 28.87 | 45.60 | 29.45 |
| 7 | 23.17 | 25.02 | 43.39 | 34.00 | 42.06 | 38.19 | 47.56 | 38.35 |
| 8 | 30.46 | 26.70 | 53.78 | 40.18 | 59.58 | 43.64 | 63.91 | 45.72 |
| 9 | 30.18 | 27.15 | 52.38 | 37.39 | 58.14 | 41.82 | 63.35 | 44.47 |
| 10 | 26.03 | 21.73 | 44.77 | 38.70 | 47.61 | 40.38 | 51.75 | 42.58 |
| 11 | 26.21 | 22.98 | 45.31 | 34.00 | 52.47 | 36.09 | 56.23 | 41.06 |
| 12 | 30.18 | 27.80 | 52.17 | 40.80 | 58.18 | 45.59 | 62.10 | 45.71 |
| 13 | 28.45 | 23.97 | 46.04 | 33.21 | 52.15 | 38.11 | 56.16 | 39.20 |
| 14 | 28.11 | 24.72 | 48.15 | 36.82 | 53.09 | 40.80 | 58.23 | 44.38 |
| 15 | 27.30 | 22.85 | 50.44 | 35.14 | 56.29 | 40.09 | 59.44 | 43.50 |
| 16 | 29.87 | 27.01 | 50.61 | 40.75 | 55.60 | 43.95 | 59.04 | 45.58 |
| 17 | 18.54 | 14.42 | 36.73 | 27.25 | 39.42 | 28.32 | 45.26 | 30.47 |
| 18 | 31.11 | 27.95 | 52.74 | 36.42 | 54.37 | 41.00 | 57.36 | 40.81 |
| 19 | 28.49 | 26.21 | 49.52 | 38.17 | 54.40 | 42.94 | 59.12 | 40.46 |
| 20 | 29.77 | 26.89 | 53.70 | 38.84 | 59.21 | 42.83 | 65.36 | 46.17 |
| 21 | 27.51 | 24.55 | 49.83 | 36.95 | 57.25 | 42.13 | 63.00 | 44.16 |
| 22 | 25.82 | 22.24 | 44.99 | 35.93 | 48.77 | 38.46 | 53.44 | 42.43 |
| 23 | 24.05 | 23.04 | 44.58 | 34.24 | 49.79 | 35.96 | 54.02 | 38.86 |

S2 Table C. X value of post-treatment

|  | U6C | U6R | U3C | U3R | U2C | U2R | U1C | U1R |
| --- | --- | --- | --- | --- | --- | --- | --- | --- |
| 1 | 0.01 | 17.56 | -1.43 | 21.47 | 0.39 | 18.65 | -0.06 | 21.58 |
| 2 | -0.06 | 16.01 | 0.36 | 22.95 | 1.03 | 19.06 | 0.38 | 20.94 |
| 3 | -0.03 | 14.82 | -0.10 | 17.63 | 0.68 | 14.71 | 0.18 | 17.44 |
| 4 | -0.02 | 16.16 | 0.04 | 20.58 | 1.29 | 19.64 | 0.12 | 19.24 |
| 5 | 0.02 | 15.59 | -1.17 | 21.25 | -0.41 | 18.24 | -0.09 | 20.52 |
| 6 | 0.00 | 16.93 | -0.94 | 21.77 | 0.37 | 16.80 | 0.02 | 20.47 |
| 7 | 0.01 | 20.82 | -0.76 | 22.70 | 0.29 | 17.23 | -0.03 | 18.07 |
| 8 | 0.02 | 17.66 | 0.32 | 25.48 | 0.67 | 21.13 | -0.10 | 21.33 |
| 9 | -0.02 | 18.31 | -0.31 | 23.26 | 1.64 | 19.16 | 0.15 | 20.57 |
| 10 | 0.02 | 17.09 | 0.25 | 21.26 | 0.19 | 16.40 | -0.13 | 14.28 |
| 11 | 0.01 | 17.96 | -0.40 | 20.93 | 0.03 | 19.17 | -0.07 | 16.69 |
| 12 | 0.00 | 18.10 | -0.31 | 22.80 | 0.25 | 16.86 | -0.01 | 21.29 |
| 13 | 0.04 | 17.56 | -0.14 | 23.29 | 0.19 | 17.84 | -0.21 | 19.41 |
| 14 | -0.02 | 15.07 | 0.94 | 18.21 | 1.76 | 16.79 | 0.09 | 17.70 |
| 15 | 0.02 | 16.98 | -0.10 | 20.11 | 0.58 | 17.86 | -0.11 | 18.76 |
| 16 | -0.01 | 15.62 | -1.00 | 19.80 | 0.39 | 19.38 | 0.06 | 19.88 |
| 17 | 0.01 | 17.52 | -1.32 | 20.26 | -0.15 | 17.39 | -0.06 | 19.13 |
| 18 | -0.02 | 19.53 | -0.62 | 22.55 | -0.15 | 19.06 | 0.10 | 17.98 |
| 19 | 0.01 | 19.45 | -0.59 | 21.57 | 0.01 | 19.36 | -0.07 | 19.99 |
| 20 | 0.02 | 18.42 | 0.80 | 25.41 | 0.98 | 20.11 | -0.12 | 21.86 |
| 21 | 0.24 | 18.53 | -1.08 | 23.52 | -0.33 | 19.06 | -0.78 | 20.21 |
| 22 | -0.02 | 18.34 | -0.12 | 22.30 | -0.03 | 18.57 | 0.10 | 15.28 |
| 23 | -0.03 | 17.81 | 0.21 | 21.13 | -0.16 | 17.05 | 0.20 | 16.56 |

S2 Table D. Y value of post-treatment

|  | U6C | U6R | U3C | U3R | U2C | U2R | U1C | U1R |
| --- | --- | --- | --- | --- | --- | --- | --- | --- |
| 1 | 30.41 | 28.74 | 45.41 | 35.48 | 52.54 | 43.83 | 54.91 | 43.87 |
| 2 | 26.44 | 21.26 | 41.23 | 30.13 | 46.44 | 39.73 | 48.85 | 39.66 |
| 3 | 29.19 | 23.87 | 43.95 | 31.66 | 49.39 | 39.78 | 53.54 | 42.70 |
| 4 | 29.08 | 26.35 | 45.53 | 35.05 | 51.65 | 43.00 | 56.56 | 44.70 |
| 5 | 27.92 | 24.86 | 41.94 | 31.76 | 47.53 | 38.09 | 51.86 | 40.07 |
| 6 | 16.25 | 16.36 | 31.74 | 20.78 | 37.67 | 26.54 | 40.70 | 30.71 |
| 7 | 29.65 | 27.35 | 44.38 | 33.28 | 50.03 | 39.45 | 53.80 | 38.19 |
| 8 | 35.24 | 26.22 | 50.00 | 35.99 | 56.20 | 45.81 | 60.44 | 46.47 |
| 9 | 30.10 | 26.61 | 46.41 | 33.97 | 51.54 | 42.33 | 55.44 | 43.73 |
| 10 | 25.87 | 22.85 | 42.18 | 33.29 | 46.64 | 37.79 | 49.47 | 40.26 |
| 11 | 27.53 | 21.88 | 41.05 | 29.84 | 47.57 | 36.82 | 51.41 | 38.95 |
| 12 | 31.79 | 27.99 | 45.50 | 34.80 | 51.23 | 42.92 | 55.27 | 43.41 |
| 13 | 26.05 | 22.49 | 40.57 | 30.19 | 45.58 | 36.76 | 48.71 | 38.46 |
| 14 | 30.92 | 24.53 | 44.93 | 32.01 | 49.73 | 40.11 | 54.58 | 43.29 |
| 15 | 28.67 | 26.04 | 45.30 | 31.98 | 48.84 | 41.51 | 52.56 | 43.78 |
| 16 | 30.06 | 28.53 | 46.02 | 36.01 | 51.57 | 42.94 | 56.10 | 45.30 |
| 17 | 18.35 | 16.34 | 32.21 | 20.68 | 37.65 | 27.15 | 41.84 | 29.87 |
| 18 | 30.17 | 27.18 | 45.38 | 32.33 | 50.66 | 40.60 | 54.38 | 39.03 |
| 19 | 28.55 | 25.67 | 47.27 | 34.63 | 50.90 | 40.81 | 55.09 | 41.34 |
| 20 | 32.50 | 26.82 | 49.34 | 33.82 | 56.01 | 42.63 | 59.94 | 46.16 |
| 21 | 29.24 | 27.11 | 43.45 | 33.85 | 50.21 | 44.15 | 54.55 | 42.97 |
| 22 | 25.86 | 23.74 | 42.34 | 31.67 | 46.99 | 38.07 | 49.80 | 40.09 |
| 23 | 27.04 | 20.50 | 41.41 | 29.05 | 47.80 | 35.50 | 51.52 | 37.55 |

S2 Table E. Root length of pre/post-treatment

|  | Pre-treatment | | | |  | Post-treatment | | | |
| --- | --- | --- | --- | --- | --- | --- | --- | --- | --- |
|  | U6 | U3 | U2 | U1 |  | U6 | U3 | U2 | U1 |
| 1 | 18.01 | 25.62 | 22.55 | 25.29 |  | 17.66 | 24.96 | 20.24 | 24.32 |
| 2 | 19.74 | 27.27 | 25.06 | 27.55 |  | 16.89 | 25.22 | 19.85 | 22.52 |
| 3 | 15.71 | 23.99 | 19.14 | 20.93 |  | 15.79 | 21.83 | 17.95 | 20.44 |
| 4 | 16.84 | 23.74 | 20.93 | 22.70 |  | 16.42 | 23.38 | 21.08 | 22.64 |
| 5 | 16.40 | 23.44 | 22.69 | 24.29 |  | 16.17 | 24.77 | 21.75 | 23.90 |
| 6 | 17.34 | 26.68 | 21.37 | 24.70 |  | 16.93 | 25.50 | 20.52 | 23.22 |
| 7 | 21.08 | 24.68 | 20.46 | 24.02 |  | 21.05 | 25.96 | 20.72 | 24.07 |
| 8 | 18.75 | 27.51 | 25.56 | 26.14 |  | 19.81 | 30.02 | 24.65 | 25.73 |
| 9 | 19.17 | 26.19 | 22.29 | 23.96 |  | 18.68 | 26.89 | 20.33 | 23.64 |
| 10 | 18.42 | 23.40 | 19.87 | 17.75 |  | 17.56 | 22.81 | 18.69 | 17.14 |
| 11 | 18.39 | 25.02 | 23.21 | 21.03 |  | 18.81 | 25.01 | 23.01 | 20.95 |
| 12 | 18.69 | 27.16 | 22.52 | 26.82 |  | 18.76 | 25.48 | 18.71 | 24.39 |
| 13 | 18.16 | 26.63 | 23.29 | 24.59 |  | 17.88 | 25.64 | 20.31 | 22.15 |
| 14 | 15.64 | 21.61 | 17.43 | 20.15 |  | 16.43 | 21.58 | 18.04 | 20.94 |
| 15 | 18.50 | 27.28 | 23.07 | 23.73 |  | 17.30 | 24.23 | 18.90 | 20.82 |
| 16 | 16.40 | 24.13 | 20.58 | 22.61 |  | 16.13 | 23.29 | 21.09 | 22.63 |
| 17 | 18.40 | 26.64 | 21.99 | 23.70 |  | 17.72 | 24.48 | 20.67 | 22.63 |
| 18 | 19.33 | 26.00 | 22.32 | 23.69 |  | 19.78 | 26.59 | 22.06 | 23.57 |
| 19 | 19.01 | 26.35 | 20.15 | 25.86 |  | 19.76 | 25.51 | 22.10 | 24.41 |
| 20 | 18.57 | 28.04 | 25.73 | 25.51 |  | 19.25 | 29.70 | 24.79 | 25.98 |
| 21 | 18.61 | 26.43 | 22.74 | 25.17 |  | 18.56 | 27.00 | 21.16 | 24.29 |
| 22 | 18.98 | 25.17 | 21.89 | 19.16 |  | 18.67 | 24.83 | 20.91 | 18.02 |
| 23 | 18.00 | 25.82 | 22.73 | 22.64 |  | 19.01 | 25.12 | 22.15 | 21.51 |
